# Adherence to the diet with higher protein quality reduces the risk of colorectal cancer: results from a population-based prospective study

**DOI:** 10.3389/fnut.2025.1651848

**Published:** 2025-10-01

**Authors:** Dazhan Feng, Ke Wen, Junxia Xue, Yi Xiao, Haitao Gu, Linglong Peng, Yuxiang Luo, Ling Xiang, Yaxu Wang, Dengliang Liu

**Affiliations:** ^1^Department of Gastrointestinal Surgery, The Second Affiliated Hospital of Chongqing Medical University, Chongqing, China; ^2^Department of Radiation Oncology, Senior Department of Oncology, The Fifth Medical Center of PLA General Hospital, Beijing, China; ^3^Erasmus University Medical Center, Rotterdam, Netherlands; ^4^Department of Clinical Nutrition, The Second Affiliated Hospital of Chongqing Medical University, Chongqing, China; ^5^Department of Gastrointestinal Surgery, Chongqing Jiulongpo People’s Hospital, Chongqing, China; ^6^Department of General Surgery, Xipeng Town Health Center of Jiulongpo District, Chongqing, China

**Keywords:** healthy plate protein quality index, cancer prevention, epidemiology, colorectal cancer, cohort study

## Abstract

**Background:**

Protein quantity’s link to colorectal cancer (CRC) risk is known, but protein quality’s impact on US populations remains unclear. This study fills the gap via a population - based prospective study of 101,709 American adults from the PLCO Cancer Screening Trial.

**Methods:**

From 154,887 adults aged 55–74 years at 10 US screening centers, we formed the study group. HPPQI was calculated from the DHQ. Cox regression analysis determined HRs and 95% CIs for HPPQI - CRC associations. Subgroup and sensitivity analyses identified modifiers and ensured robustness.

**Results:**

During the study period, 1100 CRC cases and 314 CRC-related deaths were documented. In our result, HPPQI was significantly negatively associated with incidence of CRC (HR Q4 vs. Q1: 0.77; 95% CI: 0.65, 0.93; *P* = 0.009 for trend), as well as mortality rate (HR Q4 vs. Q1: 0.66; 95% CI: 0.47, 0.91; *P* = 0.024 for trend). The relationships between HPPQI and the incidence and mortality of CRC were robustly supported by sensitivity analyses. Nevertheless, upon separate examination of the relationships between HPPQI and proximal colon cancer, distal colon cancer, and rectal cancer, none of these associations attained statistical significance (all *P*-values > 0.05).

**Conclusion:**

Our findings suggest focusing on higher quality of protein consumption may be an effective approach to reduce the risk of CRC in the US population.

## Introduction

In the USA, colorectal cancer (CRC) persists as a significant public health concern, with projections estimating over 152,000 new cases and 52,000 associated deaths in 2025 alone (1). This makes CRC the second most common cancer among women and the third most common among men (2). The considerable health and economic toll of CRC highlights the critical need for the development and implementation of effective prevention strategies to reduce its incidence and mortality rates.

Among modifiable lifestyle factors, diet has been recognized as a major contributor to CRC risk, accounting for more than 40% of CRC incidence and mortality (3). The role of proteins in sustaining vital activities and promoting human health is pivotal (4). Recently, the potential association between proteins and CRC has emerged as a focal point of scientific research (5–7). As the fundamental substances constituting cells and tissues, the type, source, and intake of proteins can significantly influence CRC risk (8). However, current research on the relationship between total protein intake and the risk of CRC has yielded complex and sometimes contradictory results. Some studies have indicated an association between high - protein diets and an increased risk of CRC (9). However, we found that the proteins investigated in these studies were primarily low - quality proteins rich in harmful substances such as saturated fats, cholesterol, and heterocyclic amines. These harmful substances in the proteins can promote abnormal proliferation of intestinal cells, thereby elevating the likelihood of carcinogenesis (10). In contrast, other studies have demonstrated that high- protein diets predominantly composed of high- quality proteins rich in beneficial components like fiber, antioxidants, and phytochemicals are closely related to a significant reduction in the risk of CRC (10, 11). Therefore, optimizing the protein intake structure by increasing the proportion of high-quality proteins and reducing the intake of low-quality proteins may help lower CRC risk (12). Given these discrepancies and complexities, we shifted focus to evaluating the healthy plate protein quality index (HPPQI) rather than merely focusing on the quantity of protein intake (13).

Healthy plate protein quality index is a comprehensive index that considers multiple dimensions, including protein type, source, amino acid composition, digestibility, and bioavailability, aiming to more comprehensively reflect the nutritional value and health benefits of proteins (14–16). By calculating HPPQI, researchers can more accurately assess the specific impact of different protein sources on CRC risk, thereby providing a solid basis for formulating scientific and reasonable dietary recommendations (17). In conclusion, the relationship between protein and colorectal cancer is a complex and multifaceted issue that requires a comprehensive evaluation framework. Such a framework should shift focus from merely quantifying protein intake to prioritizing the assessment of protein quality, particularly through the HPPQI, in order to thoroughly clarify the impact of protein on colorectal cancer risk. Through in-depth research on the relationship between HPPQI and CRC risk, we hope to provide more precise and effective strategies for the prevention and treatment of CRC.

As one of the first large-scale, prospective studies in the U.S. population to specifically employ the HPPQI for assessing CRC risk, this investigation systematically evaluated the relationship between protein quality and CRC outcomes among Americans aged 55–74 years. To explore potential variations in these associations by tumor anatomical location, we conducted supplementary analyses stratified by CRC subsites. Our findings offer critical insights that could guide the development of targeted prevention strategies, ultimately aiming to alleviate the significant health and economic burdens imposed by CRC in the United States.

## Materials and methods

### Study design

This study prospectively examines participants from the Prostate, Lung, Colorectal, and Ovarian (PLCO) Cancer Screening Trial, a large-scale randomized clinical trial sponsored by the National Cancer Institute (NCI) from 1993 to 2001 (18). Approximately 150,000 men and women, aged 55–74 years, were recruited from ten screening centers nationwide. Participants were randomly allocated to either routine medical care (control group) or additional cancer screenings (intervention group) (19). The trial’s protocol received approval from institutional review boards at both the NCI and participating centers, with all participants providing informed, written consent. Detailed aspects of the PLCO trial design, including power calculations and recruitment methodologies, have been thoroughly documented in previous publications (20, 21). The PLCO trial protocol was approved by the NCI’s and participating centers’ institutional review boards, and all participants gave informed written consent.

### Data collection and covariates assessment

Within the PLCO trial, baseline demographic and lifestyle data were gathered from participants using self-administered questionnaires (Baseline Questionnaire, BQ). The key variables analyzed in our study encompassed age, sex, race, marriage, education level, smoking status, daily cigarette consumption, body mass index (BMI) at baseline, history of aspirin use, diabetes, hypertension, colorectal diverticulitis/diverticulosis, colorectal polyp and colorectal comorbidities (specifically Gardner’s syndrome, ulcerative colitis, Crohn’s disease, or familial polyposis), and family history of CRC. BMI was computed as weight (kg) divided by height squared (m^2^). Dietary intake information was collected using a validated 137-item Food Frequency Questionnaire (FFQ), termed the Dietary History Questionnaire (DHQ), which was administered 3 years post-enrollment in the PLCO trial. The DHQ evaluated portion sizes, frequencies, and types of foods and supplements consumed by participants over the preceding year. The validity of the DHQ was established through comparison with a 24-h dietary recall study (i.e., the Eating at America’s Table Study) (22). In this study, the DHQ demonstrated superior performance in assessing absolute nutrient intake compared to other commonly utilized FFQs, such as the Block and Willett questionnaires (22).

### Population for analysis

To ensure robust and unbiased estimation of protein quality-CRC risk associations, participants were further excluded based on the following criteria:

(1)    Failure to complete the Baseline Questionnaire (BQ) (*n* = 4,918)
(2)    Invalid Dietary History Questionnaire (DHQ) responses, defined as those failure to return DHQ responses, those lacking a completion date, those completed after the death date, those with a high frequency of missing responses (≥8), or those with extremely high energy intake values (the first or last percentile) (*n* = 38,462)
(3)    Have history of cancer prior to DHQ administration (*n* = 9,684)
(4)    Participants who exited from the PLCO cancer screening trial between enrollment and DHQ completion (due to outcome events, death, or loss to follow-up) (*n* = 114)

**FIGURE 1:**
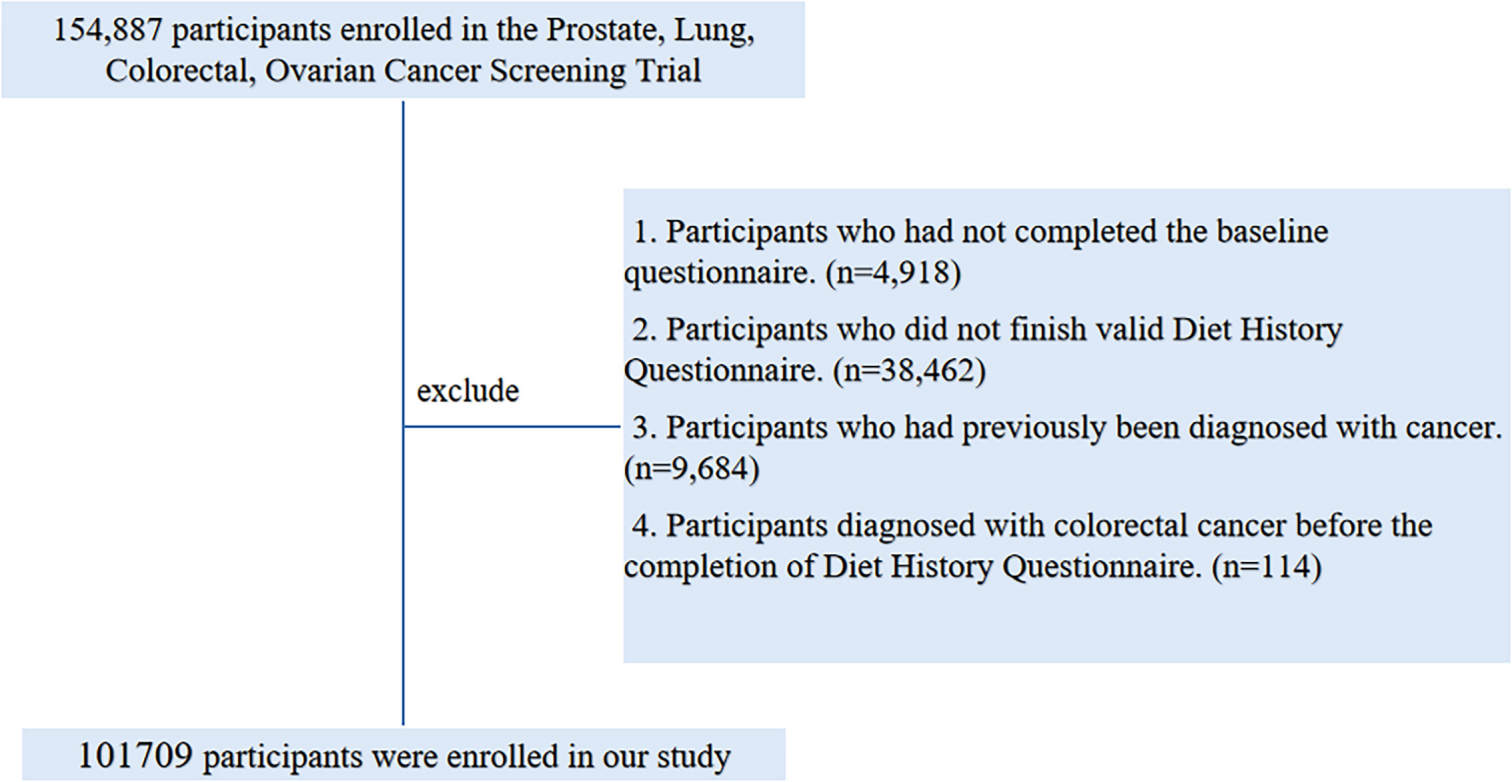
The flow chart of identifying eligible subjects. PLCO, Prostate, Lung, Colorectal, and Ovarian; BQ, Baseline Questionnaire; DHQ, Diet History Questionnaire.

Ultimately, as illustrated in Figure 1, a total of 101,709 participants met the inclusion criteria, comprising 52,250 females and 49,459 males.

### Calculation of HPPQI

Healthy plate protein quality index is established based on the nutritional protein quality recommendations derived from the latest international dietary guidelines (23, 24). To reflect differential protein bioavailability and disease associations, the numerator includes high-quality plant and animal protein sources (seafood, poultry, pulses, nuts) with favorable metabolic profiles, while the denominator comprises protein sources (red/processed meats, cheese) linked to increased CRC risk through mechanisms such as heme iron, saturated fats, and advanced glycation end products. The quality of dietary protein was determined by computing HPPQI using the following ratios (25):


HPPQI=(seafood+poultry+pulses+nuts)/

(red⁢and⁢processed⁢meats+cheese)

### Ascertainment of outcome events

In the PLCO trial, CRC case identification primarily relied on annual study update questionnaires mailed to surviving participants, which solicited information regarding any new cancer diagnoses. Self-reported CRC cases were subsequently validated through standardized medical record review, with study physicians adjudicating diagnoses in a blinded manner. Participant vital status was tracked using these annual questionnaires, with multiple follow-up attempts made to non-responders. Additional mortality surveillance involved routine linkages with the National Death Index and examination of death certificates, utilizing ICD-9 coding to ascertain causes of death.

Colorectal cancer cases in this study were classified by anatomic subsite according to International Classification of Diseases for Oncology (ICD-O2) codes, distinguishing between proximal colon (C180-C185), distal colon (C186-C187), and rectal (C199-C209) cancers. For subsite-specific analyses of colorectal cancers, cases coded as C188, C189, C212, and C218 were excluded from consideration.

### Statistical analysis

In the present analysis, covariate data exhibited varying degrees of missingness. For categorical variables with <5% missingness, such as marital status, race, education, smoking status, daily cigarette consumption, aspirin use history, history of hypertension, diabetes, colorectal diverticulitis/diverticulosis, colorectal polyps and colorectal comorbidities, and family history of cancer/CRC, missing values were imputed using the mode. Continuous variables with <5% missing data, including BMI (both baseline and change) and pack-years of cigarette smoking, were imputed using the median (26). The detailed imputation information for each missing data item and its corresponding proportion is presented in Supplementary Table 1.

**FIGURE 2:**
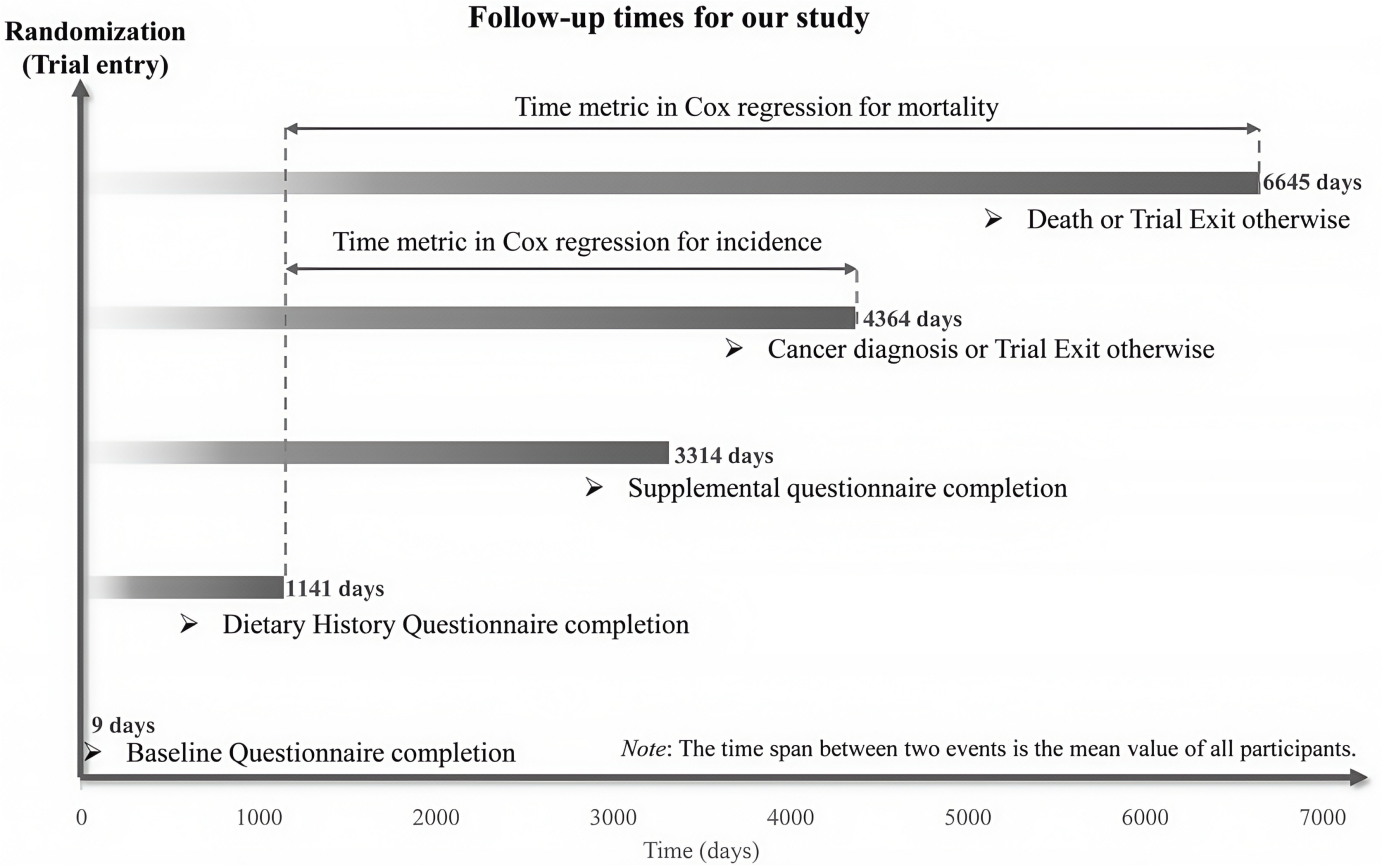
The timeline and follow-up scheme of our study.

This study defined time-to-CRC-event as the number of days from DHQ completion to CRC diagnosis or CRC-related death. Follow-up for primary outcomes extended from DHQ completion until CRC diagnosis, death, loss to follow-up, or December 31, 2009 (the end of cancer incidence follow-up), whichever occurred first. Mortality follow-up for secondary outcomes continued until 2018, as detailed in Figure 2 on the PLCO website.^1^ Cox proportional hazards models were used to estimate hazard ratios (HRs) and 95% confidence intervals (CIs) for associations between the HPPQI and outcomes, with follow-up duration serving as the time metric. HPPQI was categorized into quartiles, with a higher HPPQI category indicating better dietary protein quality. The first quartile of HPPQI was used as the referent. Additionally, continuous variables were created using quartile medians to assess linear trends, and significance was reported as *p*-values. Potential confounders included established CRC risk factors and clinically relevant variables (27). Cox models incorporated two adjustment sets: Model 1 adjusted for demographic factors (sex, age, race, education and marital status), and Model 2 further adjusted for lifestyle/clinical factors (BMI, smoking status, daily cigarette use, hypertension, diabetes, history of colorectal comorbidities, polyps and diverticulitis/diverticulosis, aspirin use and family history of CRC) and trial group. Restricted cubic spline (RCS) models were used to characterize non-linear associations between HPPQI and CRC incidence/mortality, using the median HPPQI as the reference, with non-linearity assessed by testing the regression coefficient of the second spline term against zero (28, 29). Identical analyses were conducted for CRC anatomical subsites. In addition, we employed Kaplan - Meier survival curves to describe the association between the HPPQI and the incidence/mortality rates of CRC. Moreover, we conducted a log - rank test to compare the differences in survival curves among different HPPQI level groups.

To assess potential effect modification of the relationship between HPPQI and CRC incidence and mortality across key factors, we conducted prespecified subgroup analyses. Subgroups were categorized into several categories: demographic characteristics (age > 65 vs. ≤65 years, sex male vs. female, race White vs. non-White, marital status married vs. unmarried), health conditions (diabetes status yes vs. no, hypertension status yes vs. no, baseline BMI ≤ 30 vs. >30 kg/m^2^), family and medical history (family history of CRC absent vs. present, history of colorectal diverticulitis/diverticulosis yes vs. no, history of colorectal comorbidities yes vs. no, history colorectal polyps yes vs. no), and lifestyle factors (smoking status never vs. current/former, aspirin use no vs. yes, daily cigarette consumption 0 vs. 1–20 vs. >20 cigarettes). To identify any potential spurious subgroup effects, interaction *P*-values were evaluated by comparing models with and without interaction terms.

To enhance the robustness of the findings, we conducted some sensitivity analyses (27, 30):

(1)    Excluded individuals with extreme energy intake (energy intake > 4000 kcal/day or <500 kcal/day).
(2)    Individuals with extreme BMI values (the lowest 1% and the highest 1%) were excluded.
(3)    To improve the statistical power of the study, pack-years of smoking were adjusted instead of daily cigarette consumption (ranging from 0 to 20).
(4)    Individuals with diverticulitis/diverticulosis or colorectal co-morbidity (Ulcerative colitis, Crohn’s disease, Gardner’s syndrome, or familial polyposis) were excluded.

All statistical analyses were carried out using R software version 4.3.1, with two-tailed *P* < 0.05 as the level of statistical significance.

## Result

### Participant baseline features

In this study, the median HPPQI for the participants was 0.91, with an interquartile range (IQR) of 1.13. Participants were categorized into four quartiles based on their HPPQI values: Quartile 1 (0–0.54), Quartile 2 (0.54–0.91), Quartile 3 (0.91–1.67), and Quartile 4 (>1.67). Table 1 shows that participants in Q4 (the highest quartile) were more likely to be female, non-white, and highly educated, but less likely to be married, users of aspirin, or be diagnosed with diabetes, hypertension, or polyps. Moreover, participants in Q4 are less likely to have a family history of cancer, have lower smoking intensity and have a lower and less variable BMI.

**TABLE 1:** Baseline characteristics of study population according to overall HPPQI.

		Quartiles of overall HPPQI				
Characteristics	Overall	Quartile 1	Quartile 2	Quartile 3	Quartile 4	P overall
Number of participants	10,1709	25,428	25,427	25,427	25,427	<0.001
Age	62.40 ± 5.28	62.10 ± 5.17	62.36 ± 5.24	62.51 ± 5.29	62.65 ± 5.41	
Sex						<0.001
Male	49459 (48.63%)	17470 (68.70%)	13381 (52.63%)	10515 (41.35%)	8093 (31.83%)	
Female	52250 (51.37%)	7958 (31.30%)	12046 (47.37%)	14912 (58.65%)	17334 (68.17%)	
Race						<0.001
White	94023 (92.44%)	24357 (95.79%)	23958 (94.22%)	23561 (92.66%)	22147 (87.10%)	
Non-white	7686 (7.56%)	1071 (4.21%)	1469 (5.78%)	1866 (7.34%)	3280 (12.90%)	
Education level						<0.001
College below	64923 (63.83%)	18086 (71.13%)	16553 (65.10%)	15751 (61.95%)	14533 (57.16%)	
College graduate	17841 (17.54%)	3916 (15.40%)	4413 (17.36%)	4695 (18.46%)	4817 (18.94%)	
Postgraduate	18945 (18.63%)	3426 (13.47%)	4461 (17.54%)	4981 (19.59%)	6077 (23.90%)	
Marriage						<0.001
Married	79788 (78.45%)	20442 (80.39%)	20668 (81.28%)	19998 (78.65%)	18680 (73.47%)	
Unmarried	21921 (21.55%)	4986 (19.61%)	4759 (18.72%)	5429 (21.35%)	6747 (26.53%)	
Diabetes history						<0.001
No	94907 (93.31%)	23487 (92.37%)	23683 (93.14%)	23801 (93.61%)	23936 (94.14%)	
Yes	6802 (6.69%)	1941 (7.63%)	1744 (6.86%)	1626 (6.39%)	1491 (5.86%)	
Aspirin use history						<0.001
No	53927 (53.02%)	13172 (51.80%)	13333 (52.44%)	13412 (52.75%)	14010 (55.10%)	
Yes	47782 (46.98%)	12256 (48.20%)	12094 (47.56%)	12015 (47.25%)	11417 (44.90%)	
Family history of colorectal cancer						<0.001
No	88910 (87.42%)	22183 (87.24%)	22218 (87.38%)	22302 (87.71%)	22207 (87.34%)	
Yes	10306 (10.13%)	2493 (9.80%)	2587 (10.17%)	2534 (9.97%)	2692 (10.59%)	
Possibly	2493 (2.45%)	752 (2.96%)	622 (2.45%)	591 (2.32%)	528 (2.08%)	
Diverticulitis/diverticulosis history						<0.001
No	94886 (93.29%)	23971 (94.27%)	23732 (93.33%)	23582 (92.74%)	23601 (92.82%)	
Yes	6823 (6.71%)	1457 (5.73%)	1695 (6.67%)	1845 (7.26%)	1826 (7.18%)	
Colorectal comorbidities history						0.168
No	100353 (98.67%)	25123 (98.80%)	25086 (98.66%)	25069 (98.59%)	25075 (98.62%)	
Yes	1356 (1.33%)	305 (1.20%)	341 (1.34%)	358 (1.41%)	352 (1.38%)	
Colorectal polyp history						0.031
No	94944 (93.35%)	23744 (93.38%)	23638 (92.96%)	23768 (93.48%)	23794 (93.58%)	
Yes	6765 (6.65%)	1684 (6.62%)	1789 (7.04%)	1659 (6.52%)	1633 (6.42%)	
Hypertension history						<0.001
No	68678 (67.52%)	16886 (66.41%)	17007 (66.89%)	17190 (67.61%)	17595 (69.20%)	
Yes	33031 (32.48%)	8542 (33.59%)	8420 (33.11%)	8237 (32.39%)	7832 (30.80%)	
Family history of cancer						<0.001
No	44876 (44.12%)	11525 (45.32%)	11135 (43.79%)	11229 (44.16%)	10987 (43.21%)	
Yes	56833 (55.88%)	13903 (54.68%)	14292 (56.21%)	14198 (55.84%)	14440 (56.79%)	
Arm						0.206
Intervention	51778 (50.91%)	12839 (50.49%)	12962 (50.98%)	13071 (51.41%)	12906 (50.76%)	
Control	49931 (49.09%)	12589 (49.51%)	12465 (49.02%)	12356 (48.59%)	12521 (49.24%)	
Smoking status						<0.001
No	48562 (47.75%)	10007 (39.35%)	11680 (45.94%)	13065 (51.38%)	13810 (54.31%)	
Current/former	53147 (52.25%)	15421 (60.65%)	13747 (54.06%)	12362 (48.62%)	11617 (45.69%)	
Body mass index at baseline (kg/m^2^)	27.22 ± 4.79	27.98 ± 4.72	27.56 ± 4.74	27.10 ± 4.72	26.26 ± 4.78	<0.001
Weight fluctuation^a^	2.88 ± 0.76	3.02 ± 0.74	2.94 ± 0.75	2.86 ± 0.75	2.72 ± 0.76	<0.001
Smoking pack-years	17.65 ± 26.59	24.26 ± 31.22	18.49 ± 26.68	15.33 ± 24.43	12.52 ± 21.63	<0.001
Daily cigarette consumption						<0.001
0	48666 (47.85%)	10047 (39.51%)	11697 (46.00%)	13087 (51.47%)	13835 (54.41%)	
1–20	33203 (32.65%)	8395 (33.01%)	8515 (33.49%)	8088 (31.81%)	8205 (32.27%)	
>20	19840 (19.51%)	6986 (27.47%)	5215 (20.51%)	4252 (16.72%)	3387 (13.32%)	

Descriptive statistics are presented as (mean ± standard deviation) and number (percentage) for continuous and categorical.

^a^Weight fluctuation was defined as the participant’s baseline weight minus weight at age 20.

### Association between CRC incidence and HPPQI

**FIGURE 3:**
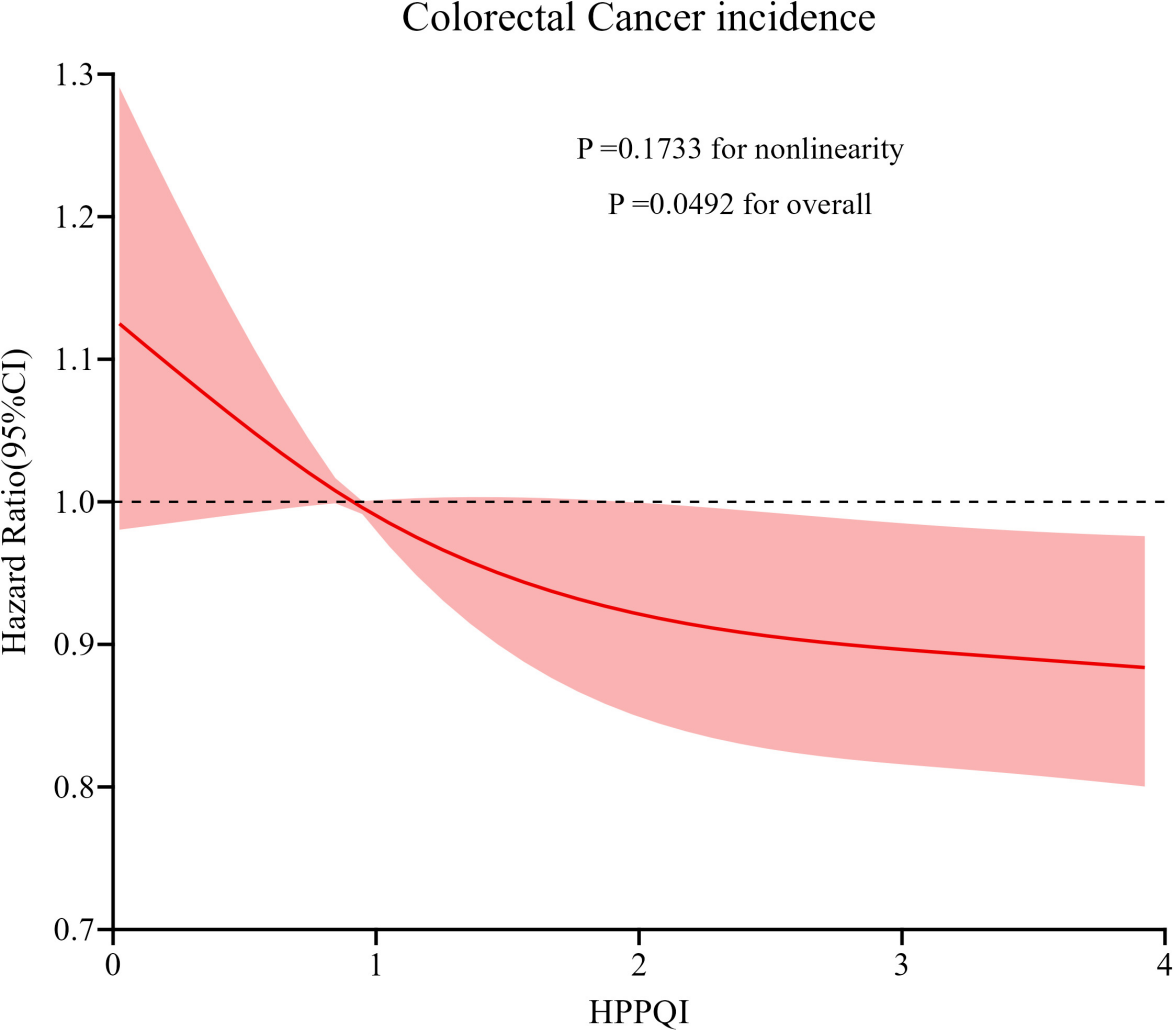
Restricted cubic spline (RCS) model on the association of HPPQI with the CRC incidence. Hazard ratio was adjusted for age (years), sex (male, female), race (white and non-white), education levels (college below, college graduate, postgraduate), marital status (married, unmarried), smoking status (never, currently/ever), number of cigarettes smoked (0, 1–20, >20 cigarettes/day), history of colorectal diverticulitis/diverticulosis (yes, no), history of colorectal comorbidities (yes, no), history of colorectal polyps (yes, no), body mass index (kg/m^2^), trail arm (intervention, control), aspirin use (yes, no), history of diabetes (yes, no), history of hypertension (yes, no) and family history of CRC (yes, no).

During the mean follow-up period of 8.82 years (896,103 person-years), a total number of 1100 CRC cases were reported, including 648 proximal colon cancer cases, 226 distal colon cancer cases and 204 rectal cancer cases, resulting in an overall incidence rate of approximately 12.28 cases per 10,000 person-years. As illustrated in Table 2, the Cox regression analysis revealed a significant negatively association between higher HPPQI values and CRC incidence after adjustment for potential confounding factors (HR Q4 vs. Q1: 0.77; 95% CI: 0.65, 0.93. *p* = 0.009 for trend). But in subsite analyses, HPPQI was not significantly associated with the incidence of proximal colon cancer, distal colon cancer or rectum cancer (all *P* > 0.05 for trend). The RCS model revealed a linear relationship between HPPQI and the risk of overall CRC incidence (Figure 3). The survival curve results indicate that there are significant differences in survival curves among different HPPQI level groups (*P* = 0.0051). As time progresses, the high - HPPQI group demonstrates a generally lower incidence of colorectal cancer overall (Supplementary Figure 1).

**TABLE 2:** Hazard ratios of the association between HPPQI and CRC incidence.

Quartiles of HPPQI	Cases	Person-years	Incidence rate per 10,000 person-years (95% confidence interval)	Hazard ratio (95% confidence interval) by HPPQI		
				Unadjusted	Model 1^a^	Model 2^b^
**Colorectal cancer**						
Quartile 1	315	220, 659.9	14.28 (12.78, 15.94)	1.000 (reference)	1.000 (reference)	1.000 (reference)
Quartile 2	274	223, 461.0	12.26 (10.89, 13.80)	0.86 (0.73, 1.01)	0.89 (0.76, 1.05)	0.90 (0.76, 1.06)
Quartile 3	272	224, 963.8	12.09 (10.74, 13.61)	0.85 (0.72, 1.00)	0.89 (0.76, 1.06)	0.89 (0.75, 1.06)
Quartile 4	239	227, 024.4	10.53 (9.28, 11.95)	0.74 (0.62, 0.87)	0.79 (0.66, 0.94)	0.77 (0.65, 0.93)
P for trend				0.001	0.017	0.009
**Proximal colon cancer**						
Quartile 1	180	220, 659.9	8.16 (7.05, 9.44)	1.000 (reference)	1.000 (reference)	1.000 (reference)
Quartile 2	162	223, 461.0	7.25 (6.22, 8.45)	0.89 (0.72, 1.10)	0.89 (0.72, 1.11)	0.88 (0.71, 1.09)
Quartile 3	159	224, 963.8	7.07 (6.05, 8.25)	0.87 (0.70, 1.07)	0.87 (0.70, 1.09)	0.87 (0.70, 1.08)
Quartile 4	147	227, 024.4	6.48 (5.51, 7.61)	0.79 (0.64, 0.99)	0.81 (0.64, 1.02)	0.80 (0.64, 1.01)
P for trend				0.061	0.104	0.107
**Distal colon cancer**						
Quartile 1	67	220, 659.9	3.04 (2.39, 3.86)	1.000 (reference)	1.000 (reference)	1.000 (reference)
Quartile 2	55	223, 461.0	2.46 (1.89, 3.20)	0.81 (0.57, 1.16)	0.87 (0.60, 1.24)	0.87 (0.60, 1.24)
Quartile 3	61	224, 963.8	2.71 (2.11, 3.48)	0.90 (0.63, 1.27)	0.99 (0.69, 1.41)	1.01 (0.70, 1.43)
Quartile 4	43	227, 024.4	1.89 (1.41, 2.55)	0.63 (0.43, 0.92)	0.70 (0.47, 1.05)	0.71 (0.47, 1.06)
P for trend				0.027	0.098	0.115
**Rectal cancer**						
Quartile 1	62	220, 659.9	2.81 (2.19, 3.60)	1.000 (reference)	1.000 (reference)	1.000 (reference)
Quartile 2	52	223, 461.0	2.33 (1.77, 3.05)	0.83 (0.57, 1.20)	0.93 (0.64, 1.35)	0.93 (0.64, 1.34)
Quartile 3	50	224, 963.8	2.22 (1.69, 2.93)	0.79 (0.55, 1.15)	0.96 (0.65, 1.40)	0.96 (0.65, 1.40)
Quartile 4	40	227, 024.4	1.76 (1.29, 2.40)	0.63 (0.42, 0.94)	0.81 (0.53, 1.23)	0.81 (0.53, 1.23)
P for trend				0.031	0.332	0.335

^a^Model 1 was adjusted with age (continuous), sex (male, female), race (white, non-white), education levels (college below, college graduate, postgraduate) and marriage (married, unmarried).

^b^Model 2 was adjusted for model 1 plus BMI at baseline (continuous), trail arm (intervention, control), smoking status (never, current or former), daily cigarette consumption (0, 1–20, >20), aspirin use (no, yes), family history of colorectal cancer (no, yes, possibly), history of hypertension (no, yes), history of diabetes (no, yes), history of colorectal diverticulitis/diverticulosis (no, yes), history of colorectal comorbidities (no, yes) and history of colorectal polyp (no, yes).

Stratified analyses assessed potential effect modification by participant characteristics (Supplementary Table 2), with the inverse association persisting across most subgroups defined by demographic characteristics (age, sex, race, marital status), health conditions (diabetes status, hypertension, baseline BMI), family and medical history (family history of CRC and history of colorectal comorbidities, polyps, and diverticulitis/diverticulosis), and lifestyle factors (aspirin use, smoking status, and daily cigarette consumption). However, none of the investigated factors emerged as a statistically significant moderator of the primary association based on tests for interaction. Additionally, the relationships between HPPQI and the incidence of CRC was robustly supported by sensitivity analyses (Supplementary Table 3). The survival curve results reveal significant differences in survival curves among different HPPQI level groups (*P* = 0.0018). As time progresses, the high - HPPQI group exhibits a generally lower mortality rate of colorectal cancer overall (Supplementary Figure 2).

### Association between CRC mortality and HPPQI

**FIGURE 4:**
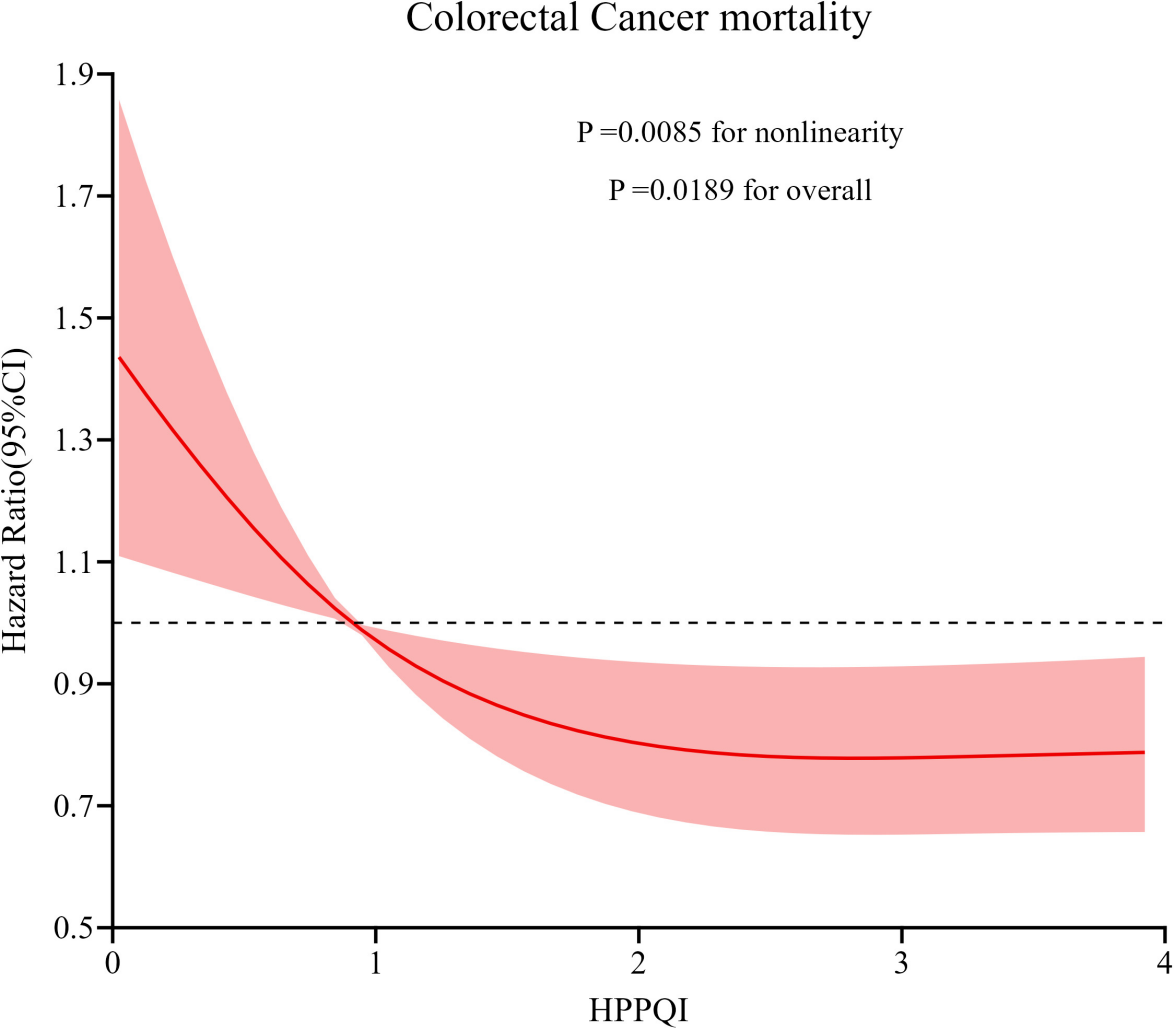
Restricted cubic spline (RCS) model on the association of HPPQI with the CRC mortality. Hazard ratio was adjusted for age (years), sex (male, female), race (white and non-white), education levels (college below, college graduate, postgraduate), marital status (married, unmarried), smoking status (never, currently/ever), number of cigarettes smoked (0, 1–20, >20 cigarettes/day), history of colorectal diverticulitis/diverticulosis (yes, no), history of colorectal comorbidities (yes, no), history of colorectal polyps (yes, no), body mass index (kg/m^2^), trail arm (intervention, control), aspirin use (yes, no), history of diabetes (yes, no), history of hypertension (yes, no) and family history of CRC (yes, no).

During the mean follow-up period of 15.07 years (1,532,681 person-years), a total number of 314 deaths attributed to CRC were identified, including 184 proximal colon cancer cases, 71 distal colon cancer cases and 41 rectal cancer cases, resulting in an overall mortality rate of approximately 2.05 cases per 10,000 person-years. As illustrated in Table 3, the Cox regression analysis revealed a significant negatively association between higher HPPQI values and CRC mortality after adjustment for potential confounding factors (HR Q4 vs. Q1: 0.66; 95% CI: 0.47, 0.91. *p* = 0.024 for trend). But in subsite analyses, HPPQI was not significantly associated with the mortality of proximal colon cancer, distal colon cancer or rectum cancer (all *P* > 0.05 for trend). The RCS model revealed a non-linear relationship between HPPQI and the risk of overall CRC mortality (Figure 4).

**TABLE 3:** Hazard ratios of the association between HPPQI and CRC mortality.

Quartiles of HPPQI	Cases	Person-years	Incidence rate per 10,000 person-years (95% confidence interval)	Hazard ratio (95% confidence interval) by HPPQI		
				Unadjusted	Model 1^a^	Model 2^b^
**Colorectal cancer**						
Quartile 1	103	369, 502.0	2.79 (2.30, 3.38)	1.000 (reference)	1.000 (reference)	1.000 (reference)
Quartile 2	81	380, 137.5	2.13 (1.71, 2.65)	0.77 (0.58, 1.03)	0.80 (0.60, 1.07)	0.81 (0.61, 1.09)
Quartile 3	67	387, 680.5	1.73 (1.36, 2.19)	0.63 (0.46, 0.85)	0.66 (0.48, 0.91)	0.69 (0.50, 0.94)
Quartile 4	63	395, 361.0	1.59 (1.25, 2.04)	0.58 (0.43, 0.80)	0.62 (0.44, 0.86)	0.66 (0.47, 0.91)
P for trend				0.002	0.011	0.024
**Proximal colon cancer**						
Quartile 1	59	369, 502.0	1.60 (1.24, 2.06)	1.000 (reference)	1.000 (reference)	1.000 (reference)
Quartile 2	48	380, 137.5	1.26 (0.95, 1.67)	0.80 (0.54, 1.17)	0.79 (0.53, 1.15)	0.80 (0.54, 1.17)
Quartile 3	38	387, 680.5	0.98 (0.71, 1.35)	0.62 (0.41, 0.94)	0.60 (0.40, 0.92)	0.62 (0.41, 0.95)
Quartile 4	39	395, 361.0	0.99 (0.72, 1.35)	0.63 (0.42, 0.95)	0.60 (0.39, 0.92)	0.64 (0.42, 0.97)
P for trend				0.044	0.040	0.068
**Distal colon cancer**						
Quartile 1	28	369, 502.0	0.76 (0.52, 1.10)	1.000 (reference)	1.000 (reference)	1.000 (reference)
Quartile 2	18	380, 137.5	0.47 (0.30, 0.75)	0.63 (0.35, 1.14)	0.72 (0.39, 1.31)	0.73 (0.40, 1.33)
Quartile 3	15	387, 680.5	0.39 (0.23, 0.64)	0.52 (0.28, 0.97)	0.64 (0.34, 1.21)	0.67 (0.35, 1.27)
Quartile 4	10	395, 361.0	0.25 (0.14, 0.47)	0.34 (0.17, 0.70)	0.45 (0.21, 0.96)	0.49 (0.23, 1.04)
P for trend				0.006	0.052	0.079
**Rectal cancer**						
Quartile 1	15	369, 502.0	0.41 (0.25, 0.67)	1.000 (reference)	1.000 (reference)	1.000 (reference)
Quartile 2	12	380, 137.5	0.32 (0.18, 0.55)	0.78 (0.36, 1.66)	0.87 (0.40, 1.86)	0.87 (0.41, 1.87)
Quartile 3	14	387, 680.5	0.36 (0.22, 0.61)	0.89 (0.43, 1.84)	1.05 (0.50, 2.21)	1.07 (0.51, 2.26)
Quartile 4	13	395, 361.0	0.33 (0.19, 0.56)	0.81 (0.39, 1.71)	0.97 (0.44, 2.14)	0.99 (0.45, 2.18)
P for trend				0.726	0.953	0.918

^a^Model 1 was adjusted with age (continuous), sex (male, female), race (white, non-white), education levels (college below, college graduate, postgraduate) and marriage (married, unmarried).

^b^Model 2 was adjusted for model 1 plus BMI at baseline (continuous), trail arm (intervention, control), smoking status (never, current or former), daily cigarette consumption (0, 1–20, >20), aspirin use (no, yes), family history of colorectal cancer (no, yes, possibly), history of hypertension (no, yes), history of diabetes (no, yes), history of colorectal diverticulitis/diverticulosis (no, yes), history of colorectal comorbidities (no, yes) and history of colorectal polyp (no, yes).

Similar to the incidence-based findings, stratified analyses were conducted to evaluate the potential effect modification by participant characteristics (Supplementary Table 4). It was observed that the inverse association persisted across the majority of subgroups. Nevertheless, based on the tests for interaction, none of the investigated factors emerged as statistically significant moderators of the primary association. Sensitivity analyses demonstrated a significant robust negative association between HPPQI value and CRC mortality rates (Supplementary Table 5).

## Discussion

Based on data from the PLCO cancer screening trial, this study aims to explore the relationships between HPPQI and CRC incidence and mortality. In this study, a total of 101,709 adult participants were selected from the PLCO trial in the United States as the research population. Our results demonstrated that higher HPPQI are associated with lower incidence of CRC. Additionally, we found a similar negative correlation between HPPQI and CRC mortality risk. These associations persisted as statistically significant after comprehensive adjustment for potential confounding factors, such as lifestyle characteristics and demographic variables. The RCS model showed that overall CRC incidence exhibited a linear negative association with HPPQI, while mortality demonstrated a non-linear negative association. The sensitivity analyses suggested the robustness of our findings.

Existing research indicates that the relationship between protein intake and CRC risk is intricate and not linearly correlated, but rather modulated by multiple factors (31–33). Adequate consumption of high-quality proteins derived from sources such as lean meat, fish, beans, nuts, and dairy products may exert a positive effect on reducing CRC risk by providing essential nutrients including amino acids, vitamins, minerals, and antioxidants (34). These nutrients can enhance immune function, facilitate cell repair and renewal, and maintain intestinal health (11). However, long-term excessive intake of animal-based high-protein foods, particularly processed meats and red meats, may increase CRC risk due to mechanisms involving carcinogenic substances (e.g., nitrites and polycyclic aromatic hydrocarbons) and elevated bile acid secretion (5, 12, 35). In contrast, plant-based proteins (e.g., beans and whole grains) typically contain fewer carcinogenic components and are rich in fiber, which helps reduce the residence time of carcinogens in the intestine and thus lowers CRC risk (8, 9). Nonetheless, there remains a lack of systematic research on the association between dietary protein quality and CRC risk. To fill this critical knowledge gap and uncover the importance of dietary protein quality, we conducted a large-scale prospective cohort study. This study comprehensively evaluated potential confounding factors and investigated the correlation between protein quality and CRC incidence and mortality. Notably, this research revealed for the first time a significant correlation between a higher HPPQI and decreased CRC incidence and mortality risks. The findings suggest that adopting a dietary pattern with a high HPPQI may be an effective strategy to reduce CRC incidence and mortality.

Healthy plate protein quality index emphasizes the quality of protein sources in the diet, encompassing diversity, completeness of essential amino acids, bioavailability, low fat and cholesterol content, as well as the abundance of other nutrients (14–16). These factors collectively determine the absorption, utilization, and benefits of protein within the human body. A high HPPQI exhibits a close correlation with low incidence and mortality rates of CRC. From a biological perspective, the observed association can be well - explained. High - quality proteins with a diverse range of sources provide a balanced profile of essential amino acids, which are the building blocks for various proteins in the body, including those involved in immune cell synthesis and function. This ensures a robust immune system that can effectively identify and eliminate abnormal cells, including precancerous cells in the intestine, thereby reducing the risk of CRC development (10, 11). The low fat and cholesterol content in high - HPPQI proteins helps maintain a healthy lipid profile in the body, reducing the risk of chronic inflammation. Chronic inflammation is a well - known risk factor for CRC as it can create an environment that promotes cell proliferation and DNA damage. By minimizing inflammation, high - HPPQI proteins contribute to a less favorable environment for tumor growth (15). Moreover, the abundance of antioxidants and anticancer compounds in some high - quality protein sources, such as certain fish and legumes, can directly neutralize free radicals in the body. Free radicals are highly reactive molecules that can cause oxidative damage to DNA, leading to mutations and potentially cancerous growth. By combating free radical damage, these compounds decrease DNA injury and inhibit the early stages of tumorigenesis (16, 36). Concurrently, high - quality protein promotes the repair and renewal of intestinal cells, preserving the integrity of the intestinal mucosa. A healthy intestinal mucosa acts as a physical barrier, preventing the entry of harmful substances and pathogens into the underlying tissues, which further reduces the risk of CRC (11). These multifaceted mechanisms act in concert, revealing the potential value of HPPQI in the prevention and treatment of CRC, and providing a scientific basis for formulating targeted preventive strategies in the future.

This study possesses several notable strengths. Firstly, our study data derived from a large prospective cohort comprising over 100,000 participants with diverse occupational backgrounds across 10 screening centers in the United States ensuring broad representativeness of the study population, and the long-term follow-up ensured the reliability of the study findings. Secondly, the prospective design of the PLCO study effectively reduced the possibility of reverse causation due to subclinical pathological conditions leading to dietary changes thereby enhancing the credibility of the observed associations. The study rigorously controlled for selection bias by ensuring comparable proportions of CRC diagnoses between excluded and included populations, further strengthening internal validity. After comprehensive adjustment for multiple potential confounders, the reliability of the study results was reinforced (37, 38). Most importantly, this study represents the first systematic investigation of the relationship between HPPQI and the incidence and mortality of CRC, providing crucial new evidence in this field. According to the study findings, a dietary pattern with a high HPPQI is associated with reduced CRC incidence and mortality, a conclusion that remains robust even after sensitivity analyses.

There are some limitations to this study that should be considered. The use of a single baseline nutritional assessment may introduce bias over time as dietary habits evolve, potentially overlooking the cumulative effects of diet on disease incidence (39). The relatively brief DHQ may underestimate dietary intake variability while baseline diet assessments reasonably reflect habitual long-term intake patterns based on nutritional tenets (26). Secondly, the possibility of residual confounding from unmeasured factors cannot be entirely excluded, as is the case with most observational studies. Thirdly, self-reported dietary questionnaires may be subject to recall bias, potentially affecting the accuracy of dietary information assessment. Lastly, the study population included a substantial number of middle-aged and elderly Americans; however, the relationship between HPPQI and CRC incidence or mortality in other regions or age groups remains unclear. Therefore, additional research is warranted to explore the prevalence of these associations across different populations and potential differences among subgroups.

## Conclusion

Based on the existing body of evidence and the intricate, interconnected dynamics among dietary patterns, physical activity levels, and body composition characteristics, diets characterized by elevated HPPQI ought to be regarded primarily as an integral part of a holistic, healthy lifestyle, rather than a solitary, modifiable risk element. In summary, our research has uncovered a notable correlation between diets with higher HPPQI and a decreased likelihood of both CRC incidence and mortality. These results shed fresh light on the potential of dietary strategies in CRC prevention and management. They furnish a solid scientific underpinning for crafting evidence - informed, long - term dietary recommendations and formulating public health initiatives aimed at curbing CRC prevalence.

## Data Availability

The original contributions presented in this study are included in this article/Supplementary material, further inquiries can be directed to the corresponding authors.

## Footnotes

1
https://cdas.cancer.gov/learn/plco/early-qx/
