# Supplementary material for: Adherence to the diet with higher protein quality reduces the risk of colorectal cancer: results from a population-based prospective study
Source: Front Nutr. 2025 Oct 1;12:1651848. doi: 10.3389/fnut.2025.1651848 (PMC12520953; doi:10.3389/fnut.2025.1651848)
Supplement: Supplementary file 1 [file Table_1.docx]

**SUPPLEMENTARY MATERIAL**

**Adherence to the diet with higher protein quality reduces the risk of colorectal cancer: Results from a population-based prospective study.**


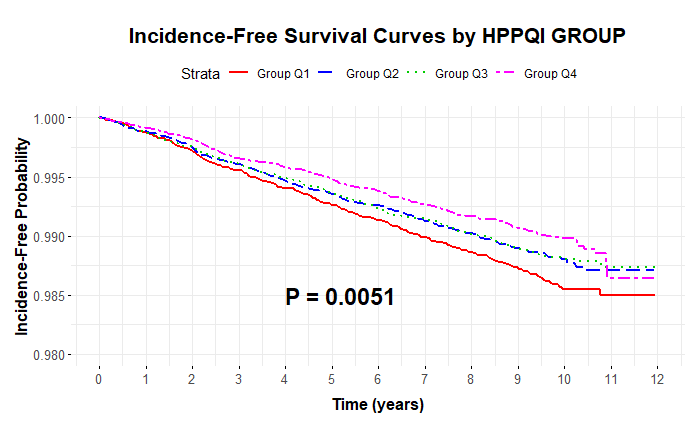


**Supplementary Figure 1**. Kaplan - Meier survival curves of HPPQI and colorectal cancer incidence and inter - group differences.


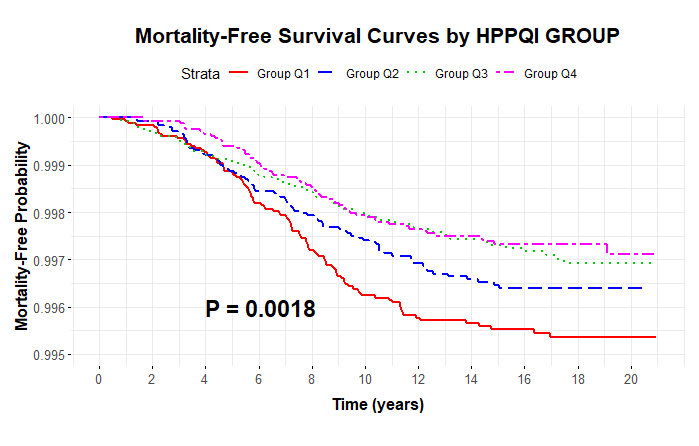


**Supplementary Figure 2**. Kaplan - Meier survival curves of HPPQI and colorectal cancer mortality and inter - group differences.

**Supplementary Table 1**. Distribution of variables with missing data before and after imputation.

| **Variable** | **Before imputation** | **After imputation** | **Number(%) with missing data** |
| --- | --- | --- | --- |
| **Race** |  |  | 37(0.04%) |
| white | 93986 (92.44%) | 94023 (92.44%) |  |
| non-white | 7686 (7.56%) | 7686 (7.56%) |  |
| **Education level** |  |  | 204(0.20%) |
| college below | 64719 (63.76%) | 64923 (63.83%) |  |
| college graduate | 17841 (17.58%) | 17841 (17.54%) |  |
| postgraduate | 18945 (18.66%) | 18945 (18.63%) |  |
| **Marriage** |  |  | 193(0.19%) |
| married | 79595 (78.41%) | 79788 (78.45%) |  |
| unmarried | 21921 (21.59%) | 21921 (21.55%) |  |
| **Diabetes history** |  |  | 538(0.53%) |
| no | 94369 (93.28%) | 94907 (93.31%) |  |
| yes | 6802 (6.72%) | 6802 (6.69%) |  |
| **Aspirin use history** |  |  | 444(0.44%) |
| no | 53483 (52.81%) | 53927 (53.02%) |  |
| yes | 47782 (47.19%) | 47782 (46.98%) |  |
| **Family history of colorectal cancer** |  |  | 781(0.77%) |
| no | 88129 (87.32%) | 88910 (87.42%) |  |
| yes | 10306 (10.21%) | 10306 (10.13%) |  |
| possibly | 2493 (2.47%) | 2493 (2.45%) |  |
| **Diverticulitis/Diverticulosis history** |  |  | 643(0.64%) |
| no | 94243 (93.25%) | 94886 (93.29%) |  |
| yes | 6823 (6.75%) | 6823 (6.71%) |  |
| **Colorectal comorbidities history** |  |  | 897(0.89%) |
| no | 99456 (98.65%) | 100353 (98.67%) |  |
| yes | 1356 (1.35%) | 1356 (1.33%) |  |
| **Colorectal polyps history** |  |  | 626(0.62%) |
| no | 94318 (93.31%) | 94944 (93.35%) |  |
| yes | 6765 (6.69%) | 6765 (6.65%) |  |
| **Hypertension history** |  |  | 513(0.51%) |
| no | 68165 (67.36%) | 68678 (67.52%) |  |
| yes | 33031 (32.64%) | 33031 (32.48%) |  |
| **Family history of cancer** |  |  | 288(0.28%) |
| no | 44588 (43.96%) | 44876 (44.12%) |  |
| yes | 56833 (56.04%) | 56833 (55.88%) |  |
| **Smoking status** |  |  | 20(0.02%) |
| no | 48542 (47.74%) | 48562 (47.75%) |  |
| current/former | 53147 (52.26%) | 53147 (52.25%) |  |
| **Body mass index at baseline (kg/m2)** | 27.23±4.82 | 27.22±4.79 | 1348(1.34%) |
| **Weight fluctuation**^a^ | 2.88±0.76 | 2.88±0.76 | 1348(1.34%) |
| **Smoking pack-years** | 17.83±26.69 | 17.65±26.59 | 1163(1.16%) |
| **Daily cigarette consumption** |  |  | 124(0.12%) |
| 0 | 48542 (47.78%) | 48666 (47.85%) |  |
| 1-20 | 33203 (32.68%) | 33203 (32.65%) |  |
| >20 | 19840 (19.53%) | 19840 (19.51%) |  |

Note: Descriptive statistics are presented as (mean ± standard deviation) and number (percentage) for continuous and categorical.

^a^ Weight fluctuation: Defined as the participant's baseline weight minus weight at age 20.

**Supplementary Table 2**. Subgroup analyses between HPPQI and CRC incidence.

| **Variables** | **Number of participates** | | **Number of cases** | |  | | **HR^b^ (95% confidence interval)** | | | | | | | **P for trend** | **P_interaction_** | | |
| --- | --- | --- | --- | --- | --- | --- | --- | --- | --- | --- | --- | --- | --- | --- | --- | --- | --- |
|  |  |  |  |  | **Quartile 1** | | **Quartile 2** | | **Quartile 3** | | **Quartile 4** | | |  |  |  |  |
| **Age(years)** |  |  | |  | |  | | | | |  | |  | | | 0.383 |  |
| <=65 | 71,841 | 642 | | 1.00 (reference) | | 0.96 (0.78, 1.18) | | 0.85 (0.68, 1.06) | | 0.78 (0.61, 0.98) | | 0.040 | | | |  |  |
| >65 | 29,868 | 458 | | 1.00 (reference) | | 0.80 (0.62, 1.05) | | 0.98 (0.76, 1.27) | | 0.84 (0.64, 1.11) | | 0.472 | | | |  |  |
| **Sex** |  |  | |  | |  | |  | |  | |  | | | | 0.892 |  |
| male | 49,459 | 608 | | 1.00 (reference) | | 0.92 (0.75, 1.13) | | 0.89 (0.72, 1.11) | | 0.82 (0.64, 1.05) | | 0.138 | | | |  |  |
| female | 52,250 | 492 | | 1.00 (reference) | | 0.83 (0.63, 1.10) | | 0.88 (0.68, 1.15) | | 0.76 (0.58, 0.99) | | 0.110 | | | |  |  |
| **Race** |  |  | |  | |  | |  | |  | |  | | | | 0.986 |  |
| white | 94,023 | 1,003 | | 1.00 (reference) | | 0.89 (0.75, 1.05) | | 0.89 (0.75, 1.06) | | 0.79 (0.66, 0.96) | | 0.027 | | | |  |  |
| non-white | 7,686 | 97 | | 1.00 (reference) | | 0.92 (0.47, 1.82) | | 1.05 (0.55, 2.01) | | 0.85 (0.46, 1.59) | | 0.563 | | | |  |  |
| **Marriage** |  |  | |  | |  | |  | |  | |  | | | | 0.655 |  |
| married | 79,788 | 855 | | 1.00 (reference) | | 0.85 (0.71, 1.02) | | 0.87 (0.73, 1.05) | | 0.75 (0.61, 0.92) | | 0.020 | | | |  |  |
| non-married | 21,921 | 245 | | 1.00 (reference) | | 1.13 (0.78, 1.64) | | 1.07 (0.74, 1.56) | | 1.00 (0.69, 1.45) | | 0.805 | | | |  |  |
| **Hypertension history** |  |  | |  | |  | |  | |  | |  | | | | 0.091 |  |
| no | 68,678 | 749 | | 1.00 (reference) | | 0.78 (0.63, 0.95) | | 0.85 (0.69, 1.03) | | 0.78 (0.63, 0.96) | | 0.126 | | | |  |  |
| yes | 33,031 | 351 | | 1.00 (reference) | | 1.17 (0.88, 1.55) | | 1.05 (0.78, 1.41) | | 0.83 (0.59, 1.15) | | 0.108 | | | |  |  |
| **Diabetes history** |  |  | |  | |  | |  | |  | |  | | | | 0.538 |  |
| no | 94,907 | 994 | | 1.00 (reference) | | 0.87 (0.73, 1.03) | | 0.89 (0.75, 1.06) | | 0.80 (0.66, 0.96) | | 0.060 | | | |  |  |
| yes | 6,802 | 106 | | 1.00 (reference) | | 1.14 (0.69, 1.88) | | 1.06 (0.62, 1.80) | | 0.74 (0.40, 1.39) | | 0.264 | | | |  |  |
| **Smoking status** |  |  | |  | |  | |  | |  | |  | | | | 0.731 |  |
| current/former | 53,147 | 615 | | 1.00 (reference) | | 0.95 (0.77, 1.17) | | 0.96 (0.77, 1.19) | | 0.84 (0.66, 1.06) | | 0.184 | | | |  |  |
| No | 48,562 | 485 | | 1.00 (reference) | | 0.81 (0.62, 1.05) | | 0.83 (0.64, 1.07) | | 0.74 (0.57, 0.96) | | 0.090 | | | |  |  |
| **Colorectal polyps history** |  |  | |  | |  | |  | |  | |  | | | | 0.521 |  |
| no | 94,944 | 1,006 | | 1.00 (reference) | | 0.88 (0.74, 1.04) | | 0.91 (0.76, 1.08) | | 0.82 (0.68, 0.99) | | 0.045 | | | |  |  |
| yes | 6,765 | 94 | | 1.00 (reference) | | 1.09 (0.63, 1.88) | | 0.89 (0.50, 1.58) | | 0.55 (0.28, 1.07) | | 0.101 | | | |  |  |
| **Colorectal comorbidities history** |  |  | |  | |  | |  | |  | |  | | | | 0.811 |  |
| no | 100,353 | 1,084 | | 1.00 (reference) | | 0.90 (0.76, 1.06) | | 0.90 (0.76, 1.07) | | 0.80 (0.67, 0.96) | | 0.037 | | | |  |  |
| yes | 1,356 | 16 | | 1.00 (reference) | | 0.68 (0.15, 3.15) | | 1.23 (0.33, 4.65) | | 0.60 (0.13, 2.81) | | 0.533 | | | |  |  |
| **Diverticulitis/Diverticulosis history** |  |  | |  | |  | |  | |  | |  | | | | 0.147 |  |
| no | 94,886 | 1,022 | | 1.00 (reference) | | 0.85 (0.72, 1.01) | | 0.88 (0.75, 1.05) | | 0.76 (0.63, 0.91) | | 0.015 | | | |  |  |
| yes | 6,823 | 78 | | 1.00 (reference) | | 1.88 (0.93, 3.79) | | 1.44 (0.70, 2.99) | | 1.69 (0.82, 3.52) | | 0.411 | | | |  |  |
| **BMI**^a^ |  |  | |  | |  | |  | |  | |  | | | | 0.483 |  |
| <=30 | 78,562 | 833 | | 1.00 (reference) | | 0.85 (0.70, 1.03) | | 0.87 (0.72, 1.05) | | 0.80 (0.65, 0.97) | | 0.113 | | | |  |  |
| >30 | 23,147 | 267 | | 1.00 (reference) | | 1.01 (0.74, 1.37) | | 1.00 (0.72, 1.39) | | 0.70 (0.47, 1.04) | | 0.053 | | | |  |  |
| **Aspirin use history** |  |  | |  | |  | |  | |  | |  | | | | 0.978 |  |
| no | 53,927 | 605 | | 1.00 (reference) | | 0.85 (0.68, 1.06) | | 0.86 (0.69, 1.08) | | 0.75 (0.59, 0.95) | | 0.060 | | | |  |  |
| yes | 47,782 | 495 | | 1.00 (reference) | | 0.95 (0.74, 1.20) | | 0.96 (0.75, 1.22) | | 0.85 (0.65, 1.11) | | 0.255 | | | |  |  |
| **Family history of colorectal cancer** |  |  | |  | |  | |  | |  | |  | | | | 0.207 |  |
| no | 88,910 | 937 | | 1.00 (reference) | | 0.82 (0.69, 0.98) | | 0.87 (0.73, 1.04) | | 0.76 (0.63, 0.92) | | 0.031 | | | |  |  |
| yes/possibly | 12,799 | 163 | | 1.00 (reference) | | 1.39 (0.91, 2.13) | | 1.14 (0.72, 1.80) | | 1.04 (0.64, 1.68) | | 0.649 | | | |  |  |
| **Daily cigarette consumption** |  |  | |  | |  | |  | |  | |  | | | | 0.650 |  |
| 0 | 48,666 | 491 | | 1.00 (reference) | | 0.82 (0.64, 1.06) | | 0.86 (0.67, 1.10) | | 0.75 (0.58, 0.98) | | 0.100 | | | |  |  |
| 1-20 | 33,203 | 377 | | 1.00 (reference) | | 0.99 (0.75, 1.31) | | 1.06 (0.80, 1.40) | | 0.81 (0.60, 1.11) | | 0.204 | | | |  |  |
| >20 | 19,840 | 232 | | 1.00 (reference) | | 0.90 (0.65, 1.25) | | 0.76 (0.52, 1.10) | | 0.90 (0.62, 1.33) | | 0.845 | | | |  |  |

^a^ BMI: Defined as body mass index at baseline (kg/m2).

^b^ Hazard ratio was adjusted for age (years), sex (male, female), race (white and non-white), education levels (college below, college graduate, postgraduate), marital status (married, unmarried), smoking status (never, currently/ever), number of cigarettes smoked (0, 1-20, > 20 cigarettes/day), history of colorectal diverticulitis/diverticulosis (yes, no), history of colorectal comorbidities (yes, no), history of colorectal polyps (yes, no), body mass index (kg/m2), trial arm (intervention, control), aspirin use (yes, no), history of diabetes (yes, no), history of hypertension (yes, no) and family history of CRC (yes, no).

**Supplementary Table 3** The sensitivity analyses between HPPQI and CRC incidence.

| **Categories** | **HR** ^e^ **(Quartile 4 vs Quartile 1, 95% CI)** | **p for trend** |
| --- | --- | --- |
| Exclude extreme energy intake ^a^ | 0.77 (0.63,0.93) | 0.016 |
| Exclude extreme BMI ^b^ | 0.80 (0.67,0.95) | 0.024 |
| Replace the Indicator of cigarettes smoked ^c^ | 0.80 (0.67,0.96) | 0.027 |
| Excluding patients with diverticulitis/diverticulosis or colorectal  co-morbidity ^d^ | 0.77 (0.64,0.92) | 0.013 |

^a^ Extreme energy intake was defined as energy intake >4000 kcal/day or <500 kcal/day.

^b^ BMI defined as body mass index at baseline (kg/m^2^).

^c^ Adjusting for pack-years of smoking (continuous) instead of daily cigarette consumption (0, 1-20, or >20).

^d^ Colorectal co-morbidity: Ulcerative colitis, Crohn's disease, Gardner's syndrome, or familial polyposis.

^e^ HR was adjusted for age (years), sex (male, female), race (white and non-white), education levels (college below, college graduate, postgraduate), marital status (married, unmarried), smoking status (never, currently/ever), number of cigarettes smoked (0, 1-20, > 20 cigarettes/day), history of colorectal diverticulitis/diverticulosis (yes, no), history of colorectal comorbidities (yes, no), history of colorectal polyps (yes, no), body mass index (kg/m2), trial arm (intervention, control), aspirin use (yes, no), history of diabetes (yes, no), history of hypertension (yes, no) and family history of CRC (yes, no).

**Supplementary Table 4**. Subgroup analyses between HPPQI and CRC mortality.

| **Variables** | **Number of participates** | | **Number of cases** | |  | | **HR^b^ (95% confidence interval)** | | | | | | | **P for trend** | **P_interaction_** | | |
| --- | --- | --- | --- | --- | --- | --- | --- | --- | --- | --- | --- | --- | --- | --- | --- | --- | --- |
|  |  |  |  |  | **Quartile 1** | | **Quartile 2** | | **Quartile 3** | | **Quartile 4** | | |  |  |  |  |
| **Age(years)** |  |  | |  | |  | | | | |  |  |  | | | 0.061 |  |
| <=65 | 71,841 | 168 | | 1.00 (reference) | | 0.84 (0.57, 1.25) | | 0.53 (0.33, 0.84) | | 0.76 (0.49, 1.17) | | | 0.407 | | |  |  |
| >65 | 29,868 | 146 | | 1.00 (reference) | | 0.79 (0.51, 1.23) | | 0.87 (0.56, 1.34) | | 0.51 (0.31, 0.86) | | | 0.024 | | |  |  |
| **Sex** |  |  | |  | |  | |  | |  | | |  | | | 0.834 |  |
| male | 49,459 | 182 | | 1.00 (reference) | | 0.84 (0.58, 1.20) | | 0.66 (0.43, 1.00) | | 0.71 (0.45, 1.12) | | | 0.144 | | |  |  |
| female | 52,250 | 132 | | 1.00 (reference) | | 0.76 (0.46, 1.26) | | 0.68 (0.41, 1.11) | | 0.56 (0.34, 0.92) | | | 0.085 | | |  |  |
| **Race** |  |  | |  | |  | |  | |  | | |  | | | 0.817 |  |
| white | 94,023 | 281 | | 1.00 (reference) | | 0.80 (0.59, 1.09) | | 0.66 (0.48, 0.92) | | 0.66 (0.46, 0.93) | | | 0.036 | | |  |  |
| non-white | 7,686 | 33 | | 1.00 (reference) | | 0.91 (0.31, 2.66) | | 0.86 (0.30, 2.47) | | 0.55 (0.19, 1.57) | | | 0.181 | | |  |  |
| **Marriage** |  |  | |  | |  | |  | |  | | |  | | | 0.100 |  |
| married | 79,788 | 230 | | 1.00 (reference) | | 0.69 (0.49, 0.97) | | 0.61 (0.42, 0.89) | | 0.67 (0.46, 0.98) | | | 0.171 | | |  |  |
| non-married | 21,921 | 84 | | 1.00 (reference) | | 1.35 (0.75, 2.42) | | 0.96 (0.51, 1.78) | | 0.59 (0.30, 1.16) | | | 0.043 | | |  |  |
| **Hypertension history** |  |  | |  | |  | |  | |  | | |  | | | 0.295 |  |
| no | 68,678 | 210 | | 1.00 (reference) | | 0.72 (0.51, 1.03) | | 0.53 (0.36, 0.79) | | 0.57 (0.38, 0.85) | | | 0.022 | | |  |  |
| yes | 33,031 | 104 | | 1.00 (reference) | | 1.05 (0.62, 1.77) | | 1.10 (0.65, 1.88) | | 0.78 (0.43, 1.44) | | | 0.465 | | |  |  |
| **Diabetes history** |  |  | |  | |  | |  | |  | | |  | | | 0.138 |  |
| no | 94,907 | 285 | | 1.00 (reference) | | 0.75 (0.55, 1.02) | | 0.62 (0.44, 0.87) | | 0.61 (0.43, 0.86) | | | 0.025 | | |  |  |
| yes | 6,802 | 29 | | 1.00 (reference) | | 1.95 (0.66, 5.79) | | 1.84 (0.61, 5.60) | | 1.03 (0.28, 3.78) | | | 0.657 | | |  |  |
| **Smoking status** |  |  | |  | |  | |  | |  | | |  | | | 0.479 |  |
| current/former | 53,147 | 180 | | 1.00 (reference) | | 0.71 (0.49, 1.05) | | 0.61 (0.40, 0.94) | | 0.68 (0.44, 1.05) | | | 0.207 | | |  |  |
| No | 48,562 | 134 | | 1.00 (reference) | | 0.98 (0.61, 1.56) | | 0.79 (0.48, 1.28) | | 0.61 (0.36, 1.02) | | | 0.055 | | |  |  |
| **Colorectal polyps history** |  |  | |  | |  | |  | |  | | |  | | | 0.283 |  |
| no | 94,944 | 284 | | 1.00 (reference) | | 0.79 (0.58, 1.08) | | 0.63 (0.45, 0.89) | | 0.62 (0.44, 0.88) | | | 0.030 | | |  |  |
| yes | 6,765 | 30 | | 1.00 (reference) | | 1.15 (0.39, 3.34) | | 1.36 (0.48, 3.85) | | 0.80 (0.25, 2.63) | | | 0.542 | | |  |  |
| **Colorectal comorbidities history** |  |  | |  | |  | |  | |  | | |  | | | 0.075 |  |
| no | 100,353 | 310 | | 1.00 (reference) | | 0.81 (0.60, 1.09) | | 0.66 (0.48, 0.91) | | 0.64 (0.46, 0.89) | | | 0.032 | | |  |  |
| yes | 1,356 | 4 | | 1.00 (reference) | | / | | / | | / | | | 0.200 | | |  |  |
| **Diverticulitis/Diverticulosis history** |  |  | |  | |  | |  | |  | | |  | | | 0.654 |  |
| no | 94,886 | 296 | | 1.00 (reference) | | 0.79 (0.58, 1.07) | | 0.66 (0.48, 0.91) | | 0.60 (0.43, 0.85) | | | 0.015 | | |  |  |
| yes | 6,823 | 18 | | 1.00 (reference) | | 1.64 (0.38, 7.01) | | 1.50 (0.35, 6.41) | | 1.78 (0.40, 7.94) | | | 0.569 | | |  |  |
| **BMI**^a^ |  |  | |  | |  | |  | |  | | |  | | | 0.236 |  |
| <=30 | 78,562 | 232 | | 1.00 (reference) | | 0.71 (0.51, 1.01) | | 0.56 (0.39, 0.81) | | 0.57 (0.39, 0.83) | | | 0.025 | | |  |  |
| >30 | 23,147 | 82 | | 1.00 (reference) | | 1.16 (0.66, 2.05) | | 1.16 (0.64, 2.11) | | 0.79 (0.38, 1.62) | | | 0.483 | | |  |  |
| **Aspirin use history** |  |  | |  | |  | |  | |  | | |  | | | 0.593 |  |
| no | 53,927 | 171 | | 1.00 (reference) | | 0.85 (0.57, 1.27) | | 0.76 (0.50, 1.16) | | 0.57 (0.36, 0.90) | | | 0.038 | | |  |  |
| yes | 47,782 | 143 | | 1.00 (reference) | | 0.78 (0.50, 1.20) | | 0.60 (0.37, 0.97) | | 0.72 (0.45, 1.16) | | | 0.290 | | |  |  |
| **Family history of colorectal cancer** |  |  | |  | |  | |  | |  | | |  | | | 0.072 |  |
| no | 88,910 | 265 | | 1.00 (reference) | | 0.69 (0.50, 0.96) | | 0.65 (0.46, 0.91) | | 0.63 (0.44, 0.90) | | | 0.083 | | |  |  |
| yes/possibly | 12,799 | 49 | | 1.00 (reference) | | 1.67 (0.82, 3.39) | | 0.92 (0.40, 2.14) | | 0.57 (0.21, 1.56) | | | 0.081 | | |  |  |
| **Daily cigarette consumption** |  |  | |  | |  | |  | |  | | |  | | | 0.523 |  |
| 0 | 48,666 | 136 | | 1.00 (reference) | | 0.97 (0.61, 1.54) | | 0.80 (0.49, 1.29) | | 0.59 (0.35, 1.00) | | | 0.048 | | |  |  |
| 1-20 | 33,203 | 98 | | 1.00 (reference) | | 0.62 (0.36, 1.08) | | 0.69 (0.40, 1.20) | | 0.65 (0.36, 1.15) | | | 0.375 | | |  |  |
| >20 | 19,840 | 80 | | 1.00 (reference) | | 0.86 (0.50, 1.47) | | 0.46 (0.23, 0.94) | | 0.78 (0.41, 1.50) | | | 0.591 | | |  |  |

^a^ BMI: Defined as body mass index at baseline (kg/m2).

^b^ Hazard ratio was adjusted for age (years), sex (male, female), race (white and non-white), education levels (college below, college graduate, postgraduate), marital status (married, unmarried), smoking status (never, currently/ever), number of cigarettes smoked (0, 1-20, > 20 cigarettes/day), history of colorectal diverticulitis/diverticulosis (yes, no), history of colorectal comorbidities (yes, no), history of colorectal polyps (yes, no), body mass index (kg/m2), trial arm (intervention, control), aspirin use (yes, no), history of diabetes (yes, no), history of hypertension (yes, no) and family history of CRC (yes, no).

**Supplementary Table 5** The sensitivity analyses between HPPQI and CRC mortality.

| **Categories** | **HR** ^e^ **(Quartile 4 vs Quartile 1, 95% CI)** | **p for trend** |
| --- | --- | --- |
| Exclude extreme energy intake ^a^ | 0.60 (0.41,0.89) | 0.025 |
| Exclude extreme BMI ^b^ | 0.63 (0.45,0.88) | 0.014 |
| Replace the Indicator of cigarettes smoked ^c^ | 0.64 (0.46,0.89) | 0.017 |
| Excluding patients with diverticulitis/diverticulosis or colorectal  co-morbidity ^d^ | 0.61 (0.43,0.86) | 0.011 |

^a^ Extreme energy intake was defined as energy intake >4000 kcal/day or <500 kcal/day.

^b^ BMI defined as body mass index at baseline (kg/m^2^).

^c^ Adjusting for pack-years of smoking (continuous) instead of daily cigarette consumption (0, 1-20, or >20).

^d^ Colorectal co-morbidity: Ulcerative colitis, Crohn's disease, Gardner's syndrome, or familial polyposis.

^e^ HR was adjusted for age (years), sex (male, female), race (white and non-white), education levels (college below, college graduate, postgraduate), marital status (married, unmarried), smoking status (never, currently/ever), number of cigarettes smoked (0, 1-20, > 20 cigarettes/day), history of colorectal diverticulitis/diverticulosis (yes, no), history of colorectal comorbidities (yes, no), history of colorectal polyps (yes, no), body mass index (kg/m2), trial arm (intervention, control), aspirin use (yes, no), history of diabetes (yes, no), history of hypertension (yes, no) and family history of CRC (yes, no).
